# Anthelminthic Activity of Assassin Bug Venom against the Blood Fluke *Schistosoma mansoni*

**DOI:** 10.3390/antibiotics9100664

**Published:** 2020-10-01

**Authors:** Miray Tonk, Andreas Vilcinskas, Christoph G. Grevelding, Simone Haeberlein

**Affiliations:** 1 Institute for Insect Biotechnology, Justus Liebig University of Giessen, Heinrich-Buff-Ring 26-32, 35392 Giessen, Germany; miray.tonk@agrar.uni-giessen.de (M.T.); andreas.vilcinskas@agrar.uni-giessen.de (A.V.); 2 LOEWE Centre for Translational Biodiversity Genomics (LOEWE-TBG), Senckenberganlage 25, 60325 Frankfurt, Germany; 3 Fraunhofer Institute for Molecular Biology and Applied Ecology, Department of Bioresources, Winchester Strasse 2, 35394 Giessen, Germany; 4 Institute of Parasitology, BFS, Justus Liebig University of Giessen, Schubertstr. 81, 35392 Giessen, Germany; christoph.grevelding@vetmed.uni-giessen.de

**Keywords:** assassin bug, *Rhynocoris iracundus*, *Schistosoma mansoni*, venom, in vitro culture, natural compound, stem cells, cell proliferation

## Abstract

Helminths such as the blood fluke *Schistosoma mansoni* represent a major global health challenge due to limited availability of drugs. Most anthelminthic drug candidates are derived from plants, whereas insect-derived compounds have received little attention. This includes venom from assassin bugs, which contains numerous bioactive compounds. Here, we investigated whether venom from the European predatory assassin bug *Rhynocoris iracundus* has antischistosomal activity. Venom concentrations of 10–50 µg/mL inhibited the motility and pairing of *S. mansoni* adult worms in vitro and their capacity to produce eggs. We used EdU-proliferation assays to measure the effect of venom against parasite stem cells, which are essential for survival and reproduction. We found that venom depleted proliferating stem cells in different tissues of the male parasite, including neoblasts in the parenchyma and gonadal stem cells. Certain insect venoms are known to lyse eukaryotic cells, thus limiting their therapeutic potential. We therefore carried out hemolytic activity assays using porcine red blood cells, revealing that the venom had no significant effect at a concentration of 43 µg/mL. The observed anthelminthic activity and absence of hemolytic side effects suggest that the components of *R. iracundus* venom should be investigated in more detail as potential antischistosomal leads.

## 1. Introduction

Helminths (parasitic worms) infect more than 3.5 billion people worldwide, causing significant morbidity and economic losses [1,2]. Novel anthelminthic compounds are urgently needed to achieve better control of this important group of parasites given the limited availability of effective vaccines and drugs [3,4,5]. Among helminths, blood flukes (schistosomes) such as *Schistosoma mansoni* cause schistosomiasis, a neglected tropical disease that globally affects more than 200 million people and causes 200,000 deaths each year [6,7]. Male and female schistosomes mate in the blood vessels of their host and produce hundreds of eggs per day, which, if trapped in the liver, can trigger chronic diseases including liver fibrosis [6,8]. The treatment of schistosomiasis currently relies on a limited drug repertoire, with praziquantel as the current gold standard [9]. The continual use of this drug since its approval in the 1980s likely promotes emergence of resistant helminth populations, as evidenced by animal studies and human drug administration programs [10,11,12]. The discovery of alternative antischistosomal drugs is therefore a high priority in neglected tropical disease research [13].

Natural products represent a treasure trove for the discovery of new drugs, particularly novel anti-infectives. Plant-derived natural products have been extensively studied for their antischistosomal activity, whereas animal-derived compounds have received comparatively little attention [14], despite being the focus of drug discovery for various other therapeutic applications [15,16]. Only a few studies have reported on the antischistosomal activity of bee, scorpion, frog and snake venoms [17,18,19,20,21]. Venoms are injected by animals into the body of their victims using stings, spines or bites [22,23,24]. These complex fluids include proteolytic enzymes, biogenic amines, neurotoxic peptides, neurotransmitters, and compounds that bind to and disrupt the function of multiple molecular targets in the victim [25]. Assassin bugs (Reduviidae) are a family of predaceous hemipteran insects comprising ~6800 species [26]. They are known for their potent venom, which is injected via a straw-like proboscis to paralyze and liquefy other invertebrates as prey. Assassin bugs can also use their venom defensively against (mainly vertebrate) predators [25,27]. The composition and function of assassin bug venom is poorly understood, but more than 200 compounds have recently been identified in two reduviid species: *Platymeris biguttatus* L. and *Psytalla horrida* (both Hemiptera, Reduviidae) [28]. This is an important step toward the repurposing of venom toxins for biomedical applications. Here, we investigated the potential anthelminthic properties of venom from the European predatory assassin bug *Rhynocoris iracundus* against adult *S. mansoni*. We assessed the effects of the venom on parasite motility, reproduction, and cell proliferation in vitro for a cultivation period of 3 days. 

## 2. Results

### 2.1. Assassin Bug Venom Reduces Motility, Pairing, Attachment and Egg Production in S. mansoni

**Figure 1:**
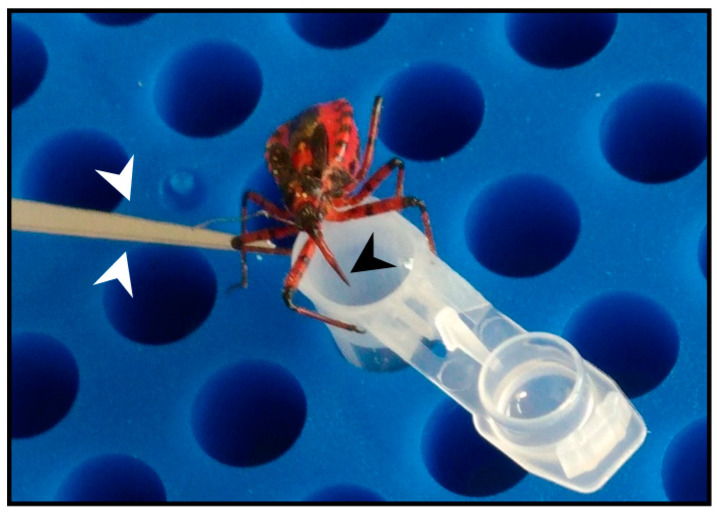
The European predatory assassin bug *Rhynocoris iracundus*. Stimulation of *R. iracundus* on the hind legs using entomological forceps (white arrow heads) encourages the insect to use its proboscis (black arrow head) to inject venom through laboratory film (Parafilm) stretched over a collection tube containing phosphate-buffered saline (PBS).

**Figure 2:**
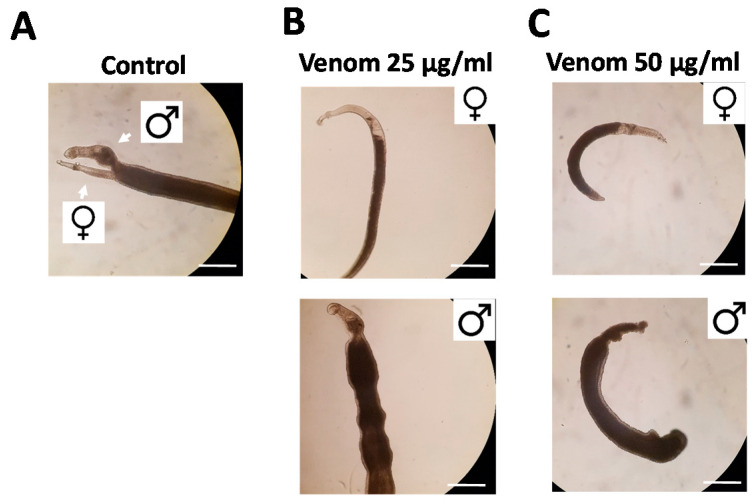
*Rhynocoris iracundus* venom affects the vitality of *Schistosoma mansoni*. Worm pairs were treated with different concentrations of venom (25 or 50 µg/mL). Representative images show worms after 72 h. (**A**) Untreated control worms remained paired and attached via their suckers to the base of the culture plate. The addition of venom at (**B**) 25 µg/mL or (**C**) 50 µg/mL induced the separation of pairs and detachment from the plate. Scale bars = 250 µm.

**Figure 3:**
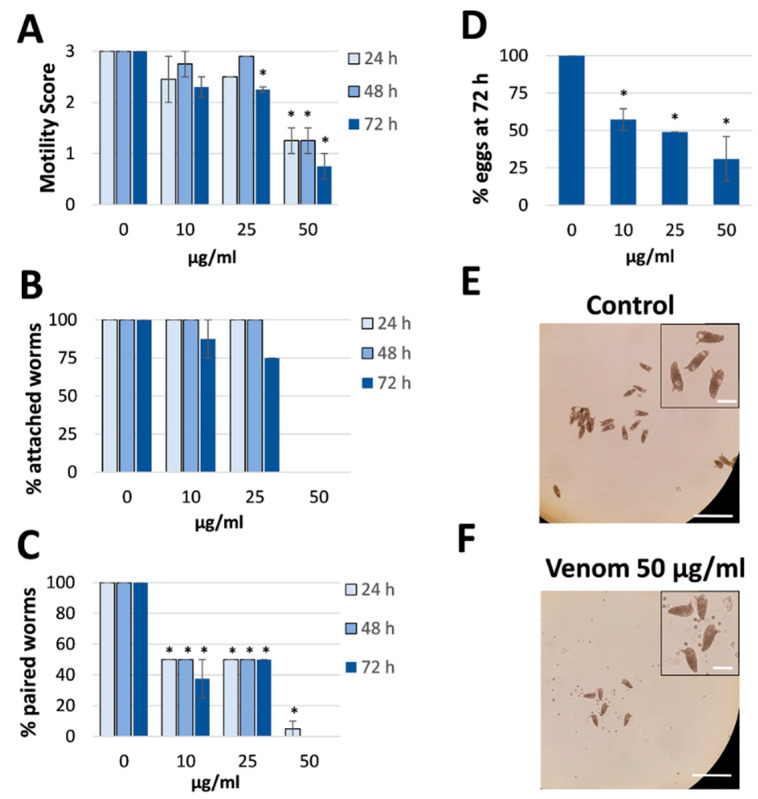
Effect of *Rhynocoris iracundus* venom on *Schistosoma mansoni* motility, pairing and egg production. Worm pairs were treated with different concentrations of venom (10–50 µg/mL) for a period of 72 h. We measured (**A**) motility, (**B**) the percentage of worms attached to the base of the plate, and (**C**) pairing stability every 24 h. (**D**) The number of eggs produced within 72 h relative to the untreated control. The shape of the eggs appeared normal after venom treatment (inserts). Graphs show a summary of two experiments with 5–8 worm pairs (mean ± SEM). Significant differences vs. the control are indicated (* *p* < 0.05, Wilcoxon rank sum test). (**E**,**F**) Representative images showing the number of eggs produced by untreated control worms and venom-treated worms (50 µg/mL). Scale bars = 250 µm, for inserts = 60 µm.

Venom was collected from *R. iracundus* by physical stimulation (Figure 1). The venom was tested for its anthelminthic activity against pairs of adult *S. mansoni* using an in vitro culture system over a period of 72 h. To assess the vitality of the worms, we determined their motility and the percentage of worms fit enough to (a) maintain the pairing state and (b) attach via their suckers to the base of the culture plate. As a positive control, worm couples were treated with different concentrations of praziquantel which caused death to all worms at 5 µM (Supplementary Figure S1). While pairs of worms in the control group remained motile and attached, those treated with 25 or 50 µg/mL of venom showed an overall loss of vitality (Figure 2, Videos S1–S4). Both males and females treated with the high dose of venom also became stunted (Figure 2C). At a venom concentration of 25 µg/mL, the motility of worms was significantly inhibited after 72 h, with male worms often being more affected (motility score 1) than females (scores 1 or 2). At the higher venom concentration (50 µg/mL), a significant loss of motility was observed already after 24 h (Figure 3A). Some (25 µg/mL) or all (50 µg/mL) worms were unable to attach to the base of the culture plate or maintain their pairing status (Figure 3B,C). Finally, a dose-dependent reduction in egg production was observed, while the shape of eggs appeared normal (Figure 3D–F). The lowest tested concentration of venom (10 µg/mL) had a slight impact on motility in some worms, but significantly reduced pairing stability and egg production (Figure 3C,D). Taken together, these results confirmed that *R. iracundus* venom affects *S. mansoni* motility, pairing stability, attachment and fecundity, starting at concentrations as low as 10 µg/mL.

### 2.2. Proliferating Stem Cells Are Depleted by Assassin Bug Venom

**Figure 4:**
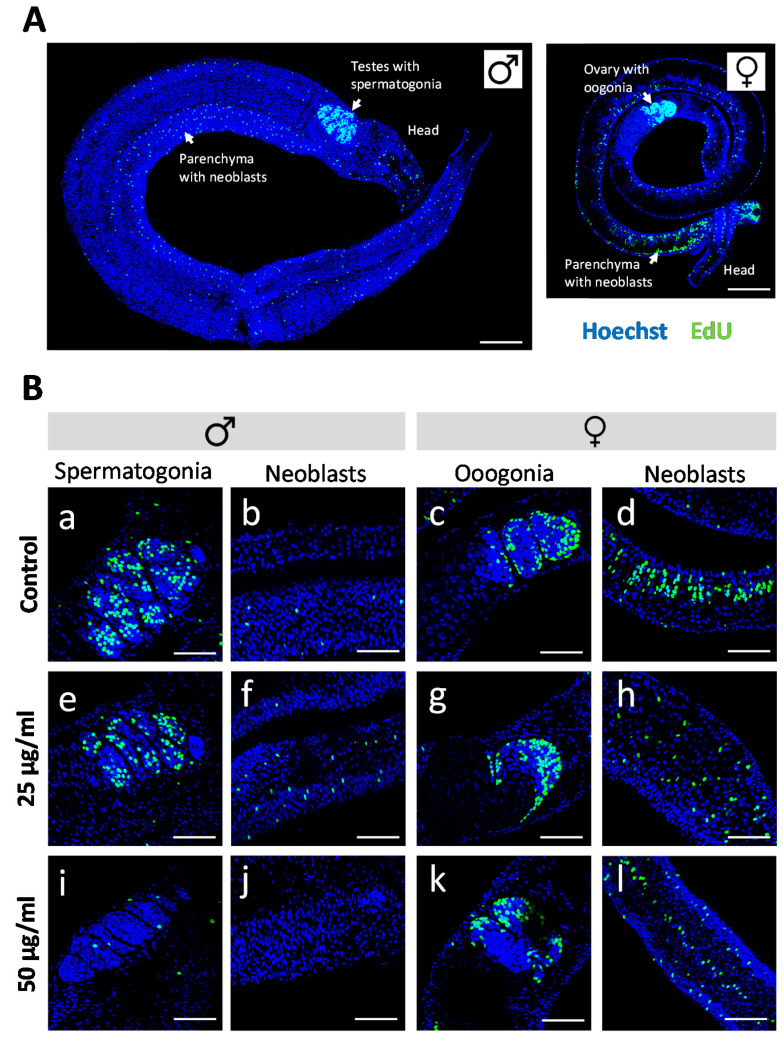
Effect of *Rhynocoris iracundus* venom on the proliferation of *Schistosoma mansoni* stem cells. (**A**) Overview of the location of parenchymal stem cells (neoblasts) and gonadal stem cells (spermatogonia and oogonia) in male and female worms. Stem cells are labeled with EdU (green), and nuclei are counterstained with Hoechst 33342 (blue). Scale bars = 100 µm. (**B**) Worm pairs were treated for 72 h with 25 or 50 µg/mL of venom or cultured without venom as a control. EdU was added during the final 24-h period. The abundance of EdU-positive proliferating stem cells was comparable in worms of the control group (a–d) and those treated with 25 µg/mL of venom (e–h) whereas 50 µg/mL of venom reduced the number of proliferating stem cells in males (i, j) but not in females (k, l). Scale bars = 50 µm. Representative images of four worms per treatment group are shown.

**Figure 5:**
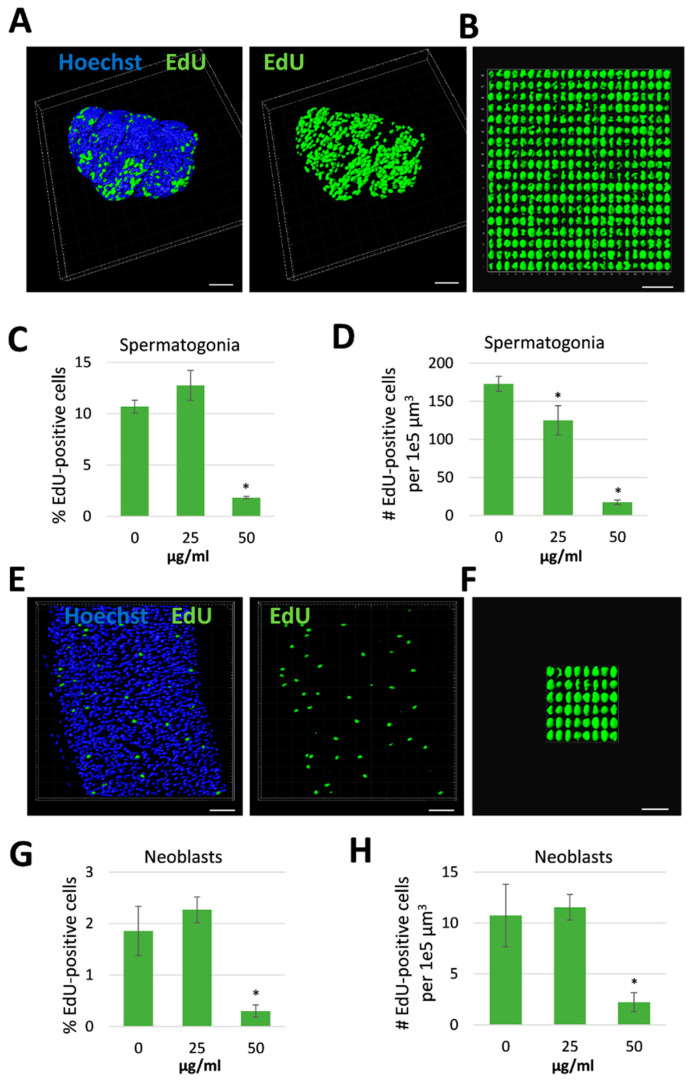
Reduction in stem cell frequency and density in male *Schistosoma mansoni* treated with *Rhynocoris iracundus* venom. Worm pairs were treated for 72 h with 25 or 50 µg/mL of venom or cultured without venom as a control. Proliferating stem cells were labeled with EdU and nuclei of all cells with Hoechst 33342. Cell numbers were quantified in z-stacks using the software package “IMARIS for cell biologists” (Bitplane). The percentage of EdU-positive cells related to the total cell number (C, G) and the number of EdU-positive cells per 1e5 µm^3^ tissue were calculated (D, H). (**A**) Representative images of testes from one worm which was digitally separated from the surrounding tissue using IMARIS. Nuclei are depicted in blue, stem cells in green. Scale bar = 40 µm. (**B**) All EdU-positive stem cells (spermatogonia) from the testes shown in (A) were aligned and quantified. Scale bar = 25 µm. The frequency (**C**) and density (**D**) of spermatogonial stem cells in testes were calculated. (**E**) Representative images of parenchyma from one worm after processing with IMARIS. Nuclei are depicted in blue, stem cells in green. Scale bar = 30 µm. (**F**) All EdU-positive stem cells (neoblasts) from the parenchymatic area shown in (E) were aligned and quantified. Scale bar = 15 µm. The frequency (**G**) and density (**H**) of neoblasts were calculated. Four worms per treatment group were analyzed. Statistical differences compared to the untreated group are indicated with * *p* < 0.05 (Wilcoxon rank sum test).

Antischistosomal effects may be associated with a decrease in the number of proliferating stem cells [29], which are considered essential for parasite development and survival [30]. We therefore investigated whether *R. iracundus* venom had a similar effect. Because stem cells are the only proliferating cells in adult schistosomes [31], we made use of the thymidine analog EdU (5-ethynyl-2-deoxyuridine) in order to visualize proliferating stem cells in whole-mount worms. EdU-positive stem cells were observed throughout the parenchyma of male and female worms (Figure 4A). These are known as neoblasts and have been shown to provide a constant stream of new cells for the development of the tegument, gastrodermis and potentially other tissues [31]. EdU-positive stem cells were also abundant in the gonads: spermatogonia in testes and oogonia in the ovary (Figure 4A), which give rise to germ cells. The analysis of venom-treated female worms by confocal laser scanning microscopy (CLSM) revealed no obvious change in the number of EdU-positive stem cells compared to untreated controls. However, the number of proliferating stem cells in males treated with 50 µg/mL venom fell to near zero in both the parenchyma and gonads (Figure 4B). To quantify this effect, we performed 3D image analysis to determine the numbers of EdU-positive stem cells and of Hoechst-positive total cells in the testes (Figure 5A–D) and the parenchyma (Figure 5E–H). This revealed a significant reduction in the frequency of stem cells and of the density of stem cells per defined tissue volume with 50 µg/mL venom. This was observed for both, spermatogonial stem cells (Figure 5C,D) and parenchymatic neoblasts (Figure 5G,H).

**Figure 6:**
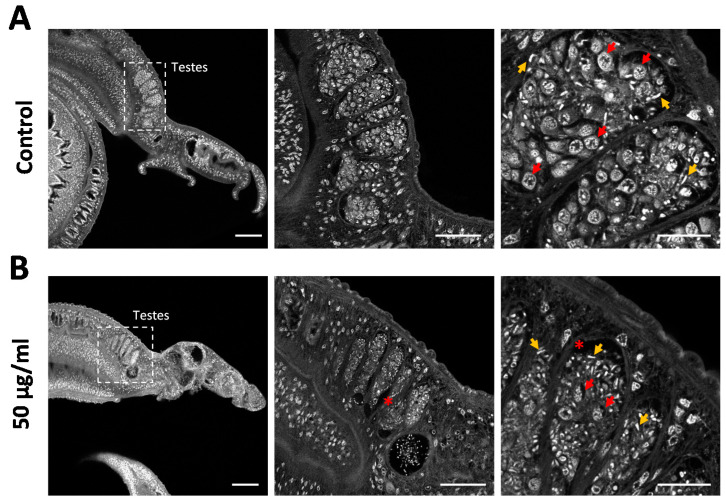
*Rhynocoris iracundus* venom reduces the number of spermatogonia in the testes of *Schistosoma mansoni.* Pairs of worms were treated with 50 µg/mL of venom for 72 h and males were stained with carmine red to reveal morphological details. (**A**) Control males feature typically pronounced testicular lobes filled with large spermatogonia (red arrows show examples) and different stages of maturing cells. Mature spermatozoa appear as small white comma-shaped cells (yellow arrows). (**B**) Testicular lobes in venom-treated males appear shrunken, include atypical cell-free areas (marked with *), and lack most of the spermatogonia, whereas mature spermatozoa are still present. The remaining spermatogonia show evidence of intracellular degradation. Scale bars = 100 µm (left), 50 µm (center), 20 µm (right).

We used carmine red staining to gain a deeper insight into the cellular composition of the testicular lobes and to assess effects on cell differentiation. Control males typically featured pronounced testicular lobes filled with a large number of large spermatogonia, various stages of maturing cells, and mature spermatozoa (Figure 6A). In contrast, the testicular lobes were shrunken after venom treatment, included atypical cell-free areas, and lacked most of the large spermatogonial stem cells. The few remaining spermatogonia showed evidence of intracellular degradation (Figure 6B). Given the abundance of spermatozoa in the lobes and seminal vesicle (Figure 6B) and the reduction of stem cell frequency and density, these results argue for the selective depletion of proliferating stem cells by assassin bug venom.

### 2.3. Hemolytic Analysis of Assassin Bug Venom

**Figure 7:**
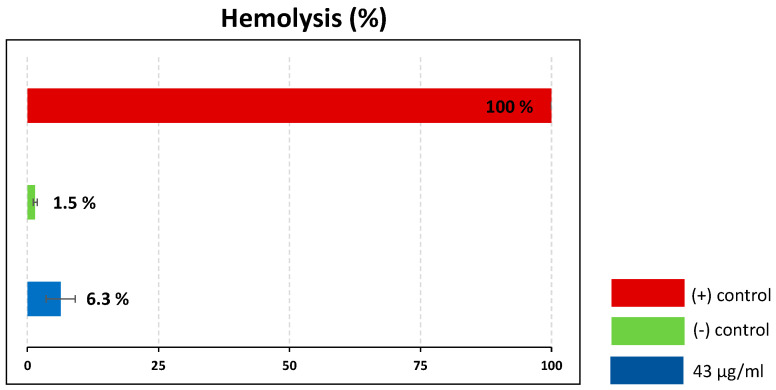
Hemolytic activity of *Rhynocoris iracundus* venom against porcine red blood cells. Relative proportion of cells lysed by *R. iracundus* venom (43 µg/mL) compared to 10% Triton X- 100 as a positive (+) control (100% lysis) and PBS as a negative (–) control.

Certain insect venoms are known for their ability to lyse eukaryotic cells, which limits their suitability as therapeutic leads [32,33]. To assess the hemolytic activity of the crude venom, we carried out hemolytic assays using porcine red blood cells, with 10% Triton X-100 as a positive control (100% lysis). The crude venom at a concentration of 43 µg/mL caused only 6.3% hemolysis, which can be regarded as non-significant (Figure 7). 

## 3. Discussion

The aim of the study was to test whether venom from *R. iracundus* has anthelminthic activity and might therefore be of interest in drug discovery research. Our data reveal that venom reduced the vitality and egg production of *S. mansoni* adult worms, which was paralleled by the depletion of proliferating stem cells in male worms.

### 3.1. Antischistosomal Effects of Assassin Bug Venom

Reduced motor activity and detachment are important antischistosomal phenotypes. In vivo, both phenotypes would very likely result in the detachment of worms from the endothelial walls of mesenteric veins and thereby the displacement and degradation of the parasite by its host. Venom clearly reduced motility and caused detachment of worms during in vitro culture. Furthermore, diminished egg production was observed, which would reduce the pathological effect of helminths in vivo because fewer eggs accumulate in the liver [6]. It is unclear whether the impairment of egg production is a direct or indirect effect of the venom. A direct effect would require venom components to interfere with pathways involved in oogenesis, as an example. However, we would argue for a rather indirect effect: when separated from their male partners, female worms arrest egg production within a few days [34]. This seems more likely because exposure to 10 µg/mL of the venom triggered the separation of mating pairs and fewer eggs were laid, but the overall fitness of most females (in terms of motility and substrate attachment) was unaffected.

The antischistosomal effects of *R. iracundus* venom are difficult to compare with other insect-derived compounds due to the sparse literature published in this field. Bee venom and bee propolis (a complex beehive product) have previously been tested in vivo in mouse models of schistosomiasis. Both products reduced the pathogen burden [21], possibly reflecting their known immunomodulatory capacity within the host [35]. However, the potential direct effects of these compounds on worm vitality were not assessed in vitro, which leaves the question unanswered whether bee-derived compounds have a direct influence on the parasite. Recently, we demonstrated a direct schistosomicidal effect for the alkaloid harmonine [29], which is produced by the harlequin ladybird *Harmonia axyridis* (Coleoptera, Coccinellidae) as a bioweapon [36]. In *S. mansoni*, harmonine not only affected motility, pairing, substrate attachment and egg laying, but also caused damage to the tegument [29], which is the physiologically active surface layer of schistosomes [37]. *R. iracundus* venom triggered mild antischistosomal effects at 10 µg/mL and severe effects at 50 µg/mL, whereas harmonine was more active, triggering mild effects at 5 µg/mL and severe effects at 10 µg/mL. It has to be taken into account that harmonine is a defined compound, whereas assassin bug venom is a complex mixture of ~220 different enzymes, toxins and other compounds [38]. In future studies, it will be important to identify the active antischistosomal components of the crude venom, and such components are likely to be active against *S. mansoni* at a much lower concentration than the crude venom.

### 3.2. Antiproliferative Effect of Assassin Bug Venom

The importance of stem cells for growth and development has been demonstrated in various helminths, including *S. mansoni* [30,39]. Compounds affecting stem cell proliferation and hence the viability of schistosomes are therefore attractive drug candidates. The venom of *R. iracundus* caused a strong depletion of proliferating stem cells in male but not in female worms. Together with the more severe reduction of motility, males appeared more sensitive to assassin bug venom compared to females. This may reflect the fact that paired females are mostly shielded from the environment, here the culture medium containing venom, by the male’s body. However, we find this unlikely because one early effect of the venom is to cause pair separation, which would expose females to the venom after ~24 h. A more plausible explanation for these phenotypes is based on sex-dependent differences in the efficiency of uptake and/or mode of action of the venom. Interestingly, lady-beetle-derived harmonine also impaired stem cell proliferation, but it affected both sexes. Enzyme activity assays suggested this may involve the inhibition of a schistosome acetylcholine esterase [29]. It is unclear whether the depletion of EdU-positive cells by harmonine reflects cell cycle arrest or cell death among the stem cell population. Our experiments with *R. iracundus* venom suggest that EdU-positive cell depletion is not based on an arrest in cell differentiation because differentiated spermatozoa were still present. Schistosome stem cells appear more sensitive towards venom than other cells, indicating that the mechanism of action targets proliferating rather than quiescent cells.

The available literature indicates a double-edged effect of animal-derived venom components on the proliferation of various cell types. Either cell proliferation was promoted, as reported for cobra, scorpion and lizard venom components tested against embryonic stem cells and mesenchymal stem cells [40,41], or venom components inhibited proliferation, as demonstrated for bufalin (a steroid hormone) and bombesin (a peptide hormone) isolated from toad venom and tested against stem cells [42,43]. Hormones may also be responsible for the anti-proliferative effect of *R. iracundus* venom. In addition, venom necrotoxins and cytotoxins might be involved, both of which typically kill cells [44]. Redulysins have been found in the venoms of other assassin bugs and were defined as putative pore-forming proteins with a cytolytic motif [45,46]. Therefore, we assume that *R. iracundus* redulysins may play a role for the observed cytotoxic effects against schistosome stem cells, with support from other compounds. 

### 3.3. Venom as Source for Antischistosomal Compounds

Results of the hemolytic assay indicated that the crude venom is not hemolytic, and from this perspective appears suitable for biotechnological applications and for the development of therapeutic leads. The absence of hemolytic activity is particularly important in the context of antischistosomal drugs, which must be bioavailable and efficacious in the blood where schistosomes live. Once active components in *R. iracundus* venom have been identified in future studies, cytotoxicity testing against different cell lines would be crucial. Together with the characterization of EC50 values against *S. mansoni*, this will allow for judging whether the selectivity is suitable to pursue venom components, e.g., to preclinical animal studies.

## 4. Materials and Methods

### 4.1. Ethical Statement

Syrian hamsters (*Mesocricetus auratus*) were used as model hosts in accordance with the European Convention for the Protection of Vertebrate Animals used for Experimental and Other Scientific Purposes (ETS No 123; revised Appendix A). The experiments were approved by the Regional Council (Regierungspraesidium) Giessen (V54-19 c 20/15 h 02 GI 18/10 Nr. A 14/2017). 

### 4.2. Production of Adult Worms

Freshwater snails of the genus *Biomphalaria glabrata* were used as the intermediate host for a Liberian strain (Bayer AG, Monheim) of *S. mansoni* [47,48]. Syrian hamsters from Janvier (France) were infected at 8 weeks of age by the paddling method [48]. In brief, hamsters were exposed to shallow water containing 1700-2000 cercariae for 45 min during which cercariae penetrated the host’s skin. Adult worm couples were collected by hepatoportal perfusion of hamsters 46 days post-infection [49]. Worms were cultured in M199 medium (Sigma-Aldrich, Germany) supplemented with 10% newborn calf serum (Sigma), 1% 1 M HEPES and 1% ABAM solution (10,000 units/mL penicillin, 10 mg/mL streptomycin and 25 mg/mL amphotericin B) at 37 °C in a 5% CO_2_ atmosphere.

### 4.3. Assassin Bug Collection and Rearing

The adult *R. iracundus* specimens were collected from North Rhine-Westphalia, Germany, with permission granted (Permission No. 425-104.1713) from the nature conservation authority (Obere Naturschutzbehörde) as part of the County Government of Rhineland-Palatinate. The insects used in this study were reared on a diet of mealworm larvae (*Tenebrio molitor* L.) in a ventilated box under constant conditions (24 ± 1 °C, 55–75% relative humidity).

### 4.4. Venom Collection 

In order to stimulate the production of venom used by *R. iracundus* for defense purposes, hind legs were gently pressed with entomological forceps to mimic a predatory attack (Figure 1). This induced the insects to display a defense posture and to penetrate laboratory film (Parafilm) stretched across the opening of a pre-cooled 200-µL Eppendorf tube containing 100 µL phosphate-buffered saline (PBS). Following this procedure, the tubes were centrifuged briefly. Four specimens of *R. iracundus* were used and venom was collected every 2–3 days. The protein content was determined using the Pierce bicinchoninic acid (BCA) assay kit (Thermo Fisher Scientific, Germany). Venom then was stored at –20 °C. 

### 4.5. Evaluation of the Physiological Effects of Venom

The anthelminthic activity of *R. iracundus* venom against adult pairs of *S. mansoni* was assessed in vitro. The worms were cultured in 96-well plates in supplemented M199 medium (one worm pair per well) mixed with different concentrations of the venom (10, 25 or 50 µg/mL) or the same volume of PBS as a negative control. The worms were incubated at 37 °C in a 5% CO_2_ atmosphere for 72 h, and the medium plus venom was refreshed every 24 h. Venom-induced effects on worm motility, pairing stability and attachment to the culture plate were assessed every 24 h using an inverted microscope (Labovert, Germany). Worm motility was scored as recommended by WHO-TDR [50], with the scores 3 (normal motility), 2 (reduced motility), 1 (minimal and sporadic movements) and 0 (no movement within 30 s was considered dead). Egg numbers per well were counted after the 72-h culture period. 

### 4.6. Proliferation Assay and CLSM

To assess the potential effect of venom on cell proliferation, EdU was added to a final concentration of 10 µM for the last 24 h of the in vitro culture period. The worms were then fixed with 4% paraformaldehyde, stained with the Click-iT Plus EdU Alexa Fluor 488 imaging kit (Thermo Fisher Scientific) and counterstained with Hoechst 33342 as previously described [29,51]. Morphological effects on testicular cells were assessed by fixing worms in AFA (66.5% ethanol, 1.1% paraformaldehyde, 2% glacial acetic acid) and staining with CertistainH carmine red (Merck, Germany) as previously described [52,53]. A TSC SP5 inverse confocal laser scanning microscope (Leica, Germany) was used for imaging. AlexaFluor488 and carmine red were excited using an argon-ion laser at 488 nm, and Hoechst at 405 nm. Optical section thickness and background signals were defined by setting the pinhole size to 1 Airy unit in the Leica LAS AF software. Z-stacks were acquired by CLSM with a step-size of 0.3 µm for quantification of EdU-positive stem cells and Hoechst-positive total cell numbers. For each worm, testes and two selected parenchymatic tissue areas were manually selected using the software package “IMARIS for cell biologists” (Bitplane, Switzerland). Cells were quantified applying the automatic surface creation of the software. To minimize background noise or counting of artifacts, a threshold was set prior to cell quantification that excluded objects <3 μm.

### 4.7. Hemolytic Activity Assay

Porcine blood was obtained from a local butcher and was mechanically treated to remove coagulants. Red blood cells were harvested by centrifugation (1500× *g*, 3 min, room temperature) and washed three times with PBS. A cell suspension was prepared with a dilution factor of 1:10 in PBS. Crude venom (final concentration 43 µg/mL) was mixed with the red blood cells (4.8 × 10^7^ cells/mL) in a 96-well plate and incubated for 1 h at 37 °C. Venom-induced hemolysis was then measured in relation to 10% Triton X-100 as a positive control (set at 100%) and PBS as a negative control [54].

### 4.8. Statistical Analysis

Homogeneity of variance was checked with Levene’s test (https://www.statskingdom.com/230var_levenes.htmL). Statistical significance was tested using the nonparametric Wilcoxon rank sum test (https://ccb-compute2.cs.uni-saarland.de/wtest/) [55]. *p* < 0.05 was considered statistically significant.

## 5. Conclusions

We have demonstrated antischistosomal effects of venom from the European predatory assassin bug *R. iracundus*. The effects included impairment of motility, pairing stability, attachment and egg production. Thus, assassin bug venom not only affects prey invertebrates but also helminths. Furthermore, the venom also caused the ablation of proliferating stem cells in male schistosomes. These phenotypes are reminiscent of the effects induced by paralytic and cytolytic assassin bug venoms used to subdue invertebrate prey [25,27]. The observed anthelminthic effects, together with the absence of hemolytic activity, warrant further studies to identify the antischistosomal components of *R. iracundus* venom and assess their suitability for therapeutic applications in the field of parasitology. The transcriptomic and proteomic data recently obtained for this venom will greatly facilitate future research in this direction [38] and provide insight into a new and underexploited resource for the development of anthelminthic drugs.

## Supplementary Materials

The following are available online at https://www.mdpi.com/2079-6382/9/10/664/s1. Figure S1: Effect of praziquantel on *Schistosoma mansoni* motility as a positive control for the in vitro culture assay. Worm pairs were treated with different concentrations of praziquantel (0.1–5 µM) for a period of 72 h. Motility was measured every 24 h and compared to DMSO-treated control worms. The graph shows a summary of two experiments with 10 worm pairs per experiment (mean ± SEM). Significant differences vs the control are indicated (* *p* < 0.05, Wilcoxon rank sum test), Video S1: *Schistosoma mansoni* pair in the control group. The male worm was attached via its suckers to the base of the culture plate and showed normal motility. The female resides within the ventral grove of the male partner. Normal motility involves whole body movements (displayed by the male in the video from 10 sec onwards), Video S2: *Schistosoma mansoni* pair after treatment with 25 µg/mL *Rhynocoris iracundus* venom for 72 h, showing reduced motility (motility score 2). The male worm was detached with its sucker from the base of the culture plate and did not show whole-body movements, Video S3: *Schistosoma mansoni* male treated with 50 µg/mL *Rhynocoris iracundus* venom for 72 h, showing severe loss of motility (little movement detected, confined to the posterior end, motility score 1), Video S4: *Schistosoma mansoni* female treated with 50 µg/mL *Rhynocoris iracundus* venom for 72 h, showing severe loss of motility (little movement detected, confined to the anterior and posterior ends, motility score 1).

## References

[1] Hotez P.J., Bundy D.A.P., Beegle K., Brooker S., Drake L., de Silva N., Montresor A., Engels D., Jukes M., Chitsulo L., Jamison D.T., Breman J.G., Measham A.R., Alleyne G., Claeson M., Evans D.B., Jha P., Mills A., Musgrove P. (2006). Helminth infections: Soil-transmitted helminth infections and schistosomiasis. Disease Control Priorities in Developing Countries.

[2] Feigin V. (2016). Global, regional, and national incidence, prevalence, and years lived with disability for 310 diseases and injuries, 1990–2015: A systematic analysis for the Global Burden of Disease Study 2015. Lancet.

[3] Newman D.J., Cragg G.M. (2012). Natural products as sources of new drugs over the 30 years from 1981 to 2010. J. Nat. Prod..

[4] Neves B.J., Andrade C.H., Cravo P.V. (2015). Natural products as leads in schistosome drug discovery. Molecules.

[5] Moser W., Schindler C., Keiser J. (2019). Drug Combinations against Soil-Transmitted Helminth Infections. Adv. Parasitol..

[6] Colley D.G., Bustinduy A.L., Secor W.E., King C.H. (2014). Human schistosomiasis. Lancet.

[7] Hotez P.J., Alvarado M., Basanez M.G., Bolliger I., Bourne R., Boussinesq M., Brooker S.J., Brown A.S., Buckle G., Budke C.M. (2014). The global burden of disease study 2010: Interpretation and implications for the neglected tropical diseases. PLoS Negl. Trop. Dis..

[8] Cheever A.W., Macedonia J.G., Mosimann J.E., Cheever E.A. (1994). Kinetics of egg production and egg excretion by *Schistosoma mansoni* and *S. japonicum* in mice infected with a single pair of worms. Am. J. Trop. Med. Hyg..

[9] Doenhoff M.J., Cioli D., Utzinger J. (2008). Praziquantel: Mechanisms of action, resistance and new derivatives for schistosomiasis. Curr. Opin. Infect. Dis..

[10] Fallon P.G., Doenhoff M.J. (1994). Drug-resistant schistosomiasis: Resistance to praziquantel and oxamniquine induced in *Schistosoma mansoni* in mice is drug specific. Am. J. Trop. Med. Hyg..

[11] Botros S.S., Bennett J.L. (2007). Praziquantel resistance. Expert Opin. Drug Discov..

[12] Mwangi I.N., Sanchez M.C., Mkoji G.M., Agola L.E., Runo S.M., Cupit P.M., Cunningham C. (2014). Praziquantel sensitivity of Kenyan *Schistosoma mansoni* isolates and the generation of a laboratory strain with reduced susceptibility to the drug. Int. J. Parasitol. Drugs Drug Resist..

[13] Cioli D., Pica-Mattoccia L., Basso A., Guidi A. (2014). Schistosomiasis control: Praziquantel forever?. Mol. Biochem. Parasitol..

[14] De Moraes J. (2015). Natural products with antischistosomal activity. Future Med. Chem..

[15] Herzig V., Cristofori-Armstrong B., Israel M.R., Nixon S.A., Vetter I., King G.F. (2020). Animal toxins-Nature’s evolutionary-refined toolkit for basic research and drug discovery. Biochem. Pharmacol..

[16] Mohamed Abd El-Aziz T., Garcia Soares A., Stockand J.D. (2019). Snake venoms in drug discovery: Valuable therapeutic tools for life saving. Toxins.

[17] El-Asmar M.F., Swelam N., Abdel Aal T.M., Ghoneim K., Hodhod S.S. (1980). Factor(s) in the venom of scorpions toxic to *Schistosoma mansoni* (intestinal belharzia) cercariae. Toxicon.

[18] Stábeli R.G., Amui S.F., Sant’Ana C.D., Pires M.G., Nomizo A., Monteiro M.C., Romão P.R., Guerra-Sá R., Vieira C.A., Giglio J.R. (2006). Bothrops moojeni myotoxin-II, a Lys49-phospholipase A2 homologue: An example of function versatility of snake venom proteins. Comp. Biochem. Physiol. C Toxicol. Pharmacol..

[19] De Moraes J., Nascimento C., Miura L.M., Leite J.R., Nakano E., Kawano T. (2011). Evaluation of the *in vitro* activity of dermaseptin 01, a cationic antimicrobial peptide, against *Schistosoma mansoni*. Chem. Biodivers..

[20] Hassan E.A., Abdel-Rahman M.A., Ibrahim M.M., Soliman M.F. (2016). In vitro antischistosomal activity of venom from the Egyptian snake *Cerastes cerastes*. Rev. Soc. Bras. Med. Trop..

[21] Mohamed A.H., Hassab El-Nabi S.E., Bayomi A.E., Abdelaal A.A. (2016). Effect of bee venom or proplis on molecular and parasitological aspects of *Schistosoma mansoni* infected mice. J. Parasit. Dis..

[22] Bailey P.C. (1986). The feeding behaviour of a sit-and wait-predator, *Ranatra dispar* (Heteroptera: Nepidae): Optimal foraging and feeding dynamics. Oecologia.

[23] Sano-Martins I.S., González C., Anjos I.V., Díaz J., Gonçalves L.R.C. (2018). Effectiveness of *Lonomia* antivenom in recovery from the coagulopathy induced by *Lonomia orientoandensis* and *Lonomia casanarensis* caterpillars in rats. PLoS Negl. Trop. Dis..

[24] Arif F., Williams M. (2020). Hymenoptera Stings (Bee, Vespids and Ants).

[25] Walker A.A., Weirauch C., Fry B.G., King G.F. (2016). Venoms of heteropteran insects: A treasure trove of diverse pharmacological toolkits. Toxins.

[26] Hwang W.S., Weirauch C. (2012). Evolutionary history of assassin bugs (insecta: Hemiptera: Reduviidae): Insights from divergence dating and ancestral state reconstruction. PLoS ONE.

[27] Edwards J.S. (1961). The action and composition of the saliva of an assassin bug *Platymeris rhadamanthus* Gaerst (Hemiptera, Reduviidae). J. Exp. Biol..

[28] Fischer G., Conceicao F.R., Leite F.P., Dummer L.A., Vargas G.D., Hubner Sde O., Dellagostin O.A., Paulino N., Paulino A.S., Vidor T. (2007). Immunomodulation produced by a green propolis extract on humoral and cellular responses of mice immunized with SuHV-1. Vaccine.

[29] Kellershohn J., Thomas L., Hahnel S.R., Grunweller A., Hartmann R.K., Hardt M., Vilcinskas A., Grevelding C.G., Haeberlein S. (2019). Insects in anthelminthics research: Lady beetle-derived harmonine affects survival, reproduction and stem cell proliferation of *Schistosoma mansoni*. PLoS Negl. Trop. Dis..

[30] Wendt G.R., Collins J.J. (2016). Schistosomiasis as a disease of stem cells. Curr. Opin. Genet. Dev..

[31] Collins J.J., Wang B., Lambrus B.G., Tharp M.E., Iyer H., Newmark P.A., Wang B., Lambrus B.G., Tharp M.E., Iyer H. (2013). Adult somatic stem cells in the human parasite *Schistosoma mansoni*. Nature.

[32] Monincová L., Budesínský M., Slaninová J., Hovorka O., Cvacka J., Voburka Z., Fucík V., Borovicková L., Bednárová L., Straka J. (2010). Novel antimicrobial peptides from the venom of the eusocial bee *Halictus sexcinctus* (Hymenoptera: Halictidae) and their analogs. Amino Acids.

[33] Mortari M.R., do Couto L.L., dos Anjos L.C., Mourão C.B., Camargos T.S., Vargas J.A., Oliveira F.N., Gati Cdel C., Schwartz C.A., Schwartz E.F. (2012). Pharmacological characterization of *Synoeca cyanea* venom: An aggressive social wasp widely distributed in the Neotropical region. Toxicon.

[34] Erasmus D.A. (1973). A comparative study of the reproductive system of mature, immature and “unisexual” female *Schistosoma mansoni*. Parasitology.

[35] Cornara L., Biagi M., Xiao J., Burlando B. (2017). Therapeutic properties of bioactive compounds from different honeybee products. Front. Pharmacol..

[36] Vilcinskas A., Stoecker K., Schmidtberg H., Rohrich C.R., Vogel H. (2013). Invasive harlequin ladybird carries biological weapons against native competitors. Science.

[37] Van Hellemond J.J., Retra K., Brouwers J.F., van Balkom B.W., Yazdanbakhsh M., Shoemaker C.B., Tielens A.G. (2006). Functions of the tegument of schistosomes: Clues from the proteome and lipidome. Int. J. Parasitol..

[38] Tonk M. (2020). Personal communication.

[39] Koziol U., Rauschendorfer T., Zanon Rodriguez L., Krohne G., Brehm K. (2014). The unique stem cell system of the immortal larva of the human parasite *Echinococcus multilocularis*. Evodevo.

[40] Zhou H., Li D., Shi C., Xin T., Yang J., Zhou Y., Hu S., Tian F., Wang J., Chen Y. (2015). Effects of Exendin-4 on bone marrow mesenchymal stem cell proliferation, migration and apoptosis *in vitro*. Sci. Rep..

[41] Miao Z., Lu Z., Luo S., Lei D., He Y., Wu H., Zhao J., Zheng L. (2018). Murine and Chinese cobra venom-derived nerve growth factor stimulate chondrogenic differentiation of BMSCs *in vitro*: A comparative study. Mol. Med. Rep..

[42] Assimakopoulos S.F., Tsamandas A.C., Georgiou C.D., Vagianos C.E., Scopa C.D. (2010). Bombesin and neurotensin exert antiproliferative effects on oval cells and augment the regenerative response of the cholestatic rat liver. Peptides.

[43] Liu J., Zhang Y., Sun S., Zhang G., Jiang K., Sun P., Zhang Y., Yao B., Sui R., Chen Y. (2019). Bufalin induces apoptosis and improves the sensitivity of human glioma stem-like sells to temozolamide. Oncol. Res..

[44] White J. (2000). Bites and stings from venomous animals: A global overview. Ther. Drug Monit..

[45] Walker A.A., Madio B., Jin J., Undheim E.A., Fry B.G., King G.F. (2017). Melt with this kiss: Paralyzing and liquefying venom of the assassin bug *Pristhesancus plagipennis* (hemiptera: Reduviidae). Mol. Cell. Proteom..

[46] Walker A.A., Robinson S.D., Undheim E.A.B., Jin J., Han X., Fry B.G., Vetter I., King G.F. (2019). Missiles of mass disruption: Composition and glandular origin of venom used as a projectile defensive weapon by the assassin bug *Platymeris rhadamanthus*. Toxins.

[47] Gönnert R. (1955). Schistosomiasis-Studien. II. Über die Eibildung bei Schistosoma mansoni und das Schicksal der Eier im Wirtsorganismus. Z. Trop. Parasitol..

[48] Dettman C.D., Higgins-Opitz S.B., Saikoolal A. (1989). Enhanced efficacy of the paddling method for schistosome infection of rodents by a four-step pre-soaking procedure. Parasitol. Res..

[49] Grevelding C.G. (1995). The female-specific W1 sequence of the Puerto Rican strain of *Schistosoma mansoni* occurs in both genders of a Liberian strain. Mol. Biochem. Parasitol..

[50] Ramirez B., Bickle Q., Yousif F., Fakorede F., Mouries M.A., Nwaka S. (2007). Schistosomes: Challenges in compound screening. Expert Opin. Drug Discov..

[51] Hahnel S., Quack T., Parker-Manuel S.J., Lu Z., Vanderstraete M., Morel M., Dissous C., Cailliau K., Grevelding C.G. (2014). Gonad RNA-specific qRT-PCR analyses identify genes with potential functions in schistosome reproduction such as SmFz1 and SmFGFRs. Front. Genet..

[52] Neves R.H., de Lamare Biolchini C., Machado-Silva J.R., Carvalho J.J., Branquinho T.B., Lenzi H.L., Hulstijn M., Gomes D.C. (2005). A new description of the reproductive system of *Schistosoma mansoni* (Trematoda: Schistosomatidae) analyzed by confocal laser scanning microscopy. Parasitol. Res..

[53] Beckmann S., Grevelding C.G. (2010). Imatinib has a fatal impact on morphology, pairing stability and survival of adult *Schistosoma mansoni in vitro*. Int. J. Parasitol..

[54] Tonk M., Pierrot C., Cabezas-Cruz A., Rahnamaeian M., Khalife J., Vilcinskas A. (2019). The *Drosophila melanogaster* antimicrobial peptides Mtk-1 and Mtk-2 are active against the malarial parasite *Plasmodium falciparum*. Parasitol. Res..

[55] Marx A., Backes C., Meese E., Lenhof H.P., Keller A. (2016). EDISON-WMW: Exact dynamic programing solution of the Wilcoxon-Mann-Whitney test. Genom. Proteom. Bioinform..

