# Supplementary figures and images for: Anthelminthic Activity of Assassin Bug Venom against the Blood Fluke Schistosoma mansoni
Source: Antibiotics (Basel). 2020 Oct 1;9(10):664. doi: 10.3390/antibiotics9100664 (PMC7599792; doi:10.3390/antibiotics9100664)

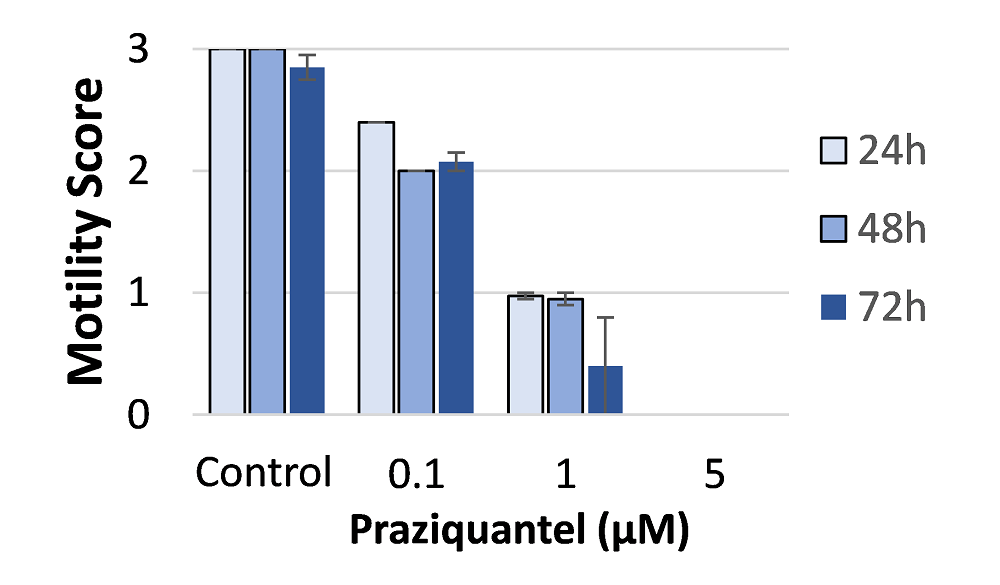

Supplement: Supplementary file 1 [file antibiotics-09-00664-s001.zip › Tonk et al., Supplementary Figure S1.tif]
